# Supplementary material for: Female Cardioprotection in a Mouse Model of Alcohol-Associated Cardiomyopathy
Source: Cells. 2025 Oct 27;14(21):1682. doi: 10.3390/cells14211682 (PMC12607819; doi:10.3390/cells14211682)
Supplement: Supplementary file 1 [file cells-14-01682-s001.zip › SupplementalTableS1-2-WayANOVA-Results.pdf]

# Supplemental Data Table 1 – Statistical Results

## Morphometric Outcomes

### Body Weight

ANOVA Summary:

| Source of Variation | p-value | Significant? |
|---|---|---|
| Interaction | ns | No |
| Sex | p<0.0001 | Yes |
| Diet_Ethanol | p=0.014 | Yes |

Tukey's Post-hoc Test:

| Comparison | Mean Difference | 95% CI of Difference | Adjusted P Value |
|---|---|---|---|
| Males:Cntl vs Males:EtOH | 3.47 | 0.75–6.20 | 0.0085 |
| Males:Cntl vs Females:Cntl | 5.37 | 2.42–8.31 | 0.0002 |
| Males:Cntl vs Females:EtOH | 6.56 | 3.97–9.14 | <0.0001 |
| Males:EtOH vs Females:Cntl | 1.89 | -1.05–4.84 | 0.3143 |
| Males:EtOH vs Females:EtOH | 3.08 | 0.50–5.67 | 0.0147 |
| Females:Cntl vs Females:EtOH | 1.19 | -1.62–4.00 | 0.6592 |

### Tibial Length

ANOVA Summary:

| Source of Variation | p-value | Significant? |
|---|---|---|
| Interaction | ns | No |
| Sex | p=0.019 | Yes |

| | | |
|---|---|---|
| Diet_Ethanol | ns | No |

Tukey's Post-hoc Test:

| Comparison | Mean Difference | 95% CI of Difference | Adjusted P Value |
|---|---|---|---|
| Males:Cntl vs Females:EtOH | 1.12 | 0.13–2.11 | 0.0215 |

### Heart Weight/Tibial Length

ANOVA Summary:

| Source of Variation | p-value | Significant? |
|---|---|---|
| Interaction | p=0.042 | Yes |
| Sex | p=0.016 | Yes |
| Diet_Ethanol | ns | No |

Tukey's Post-hoc Test:

| Comparison | Mean Difference | 95% CI of Difference | Adjusted P Value |
|---|---|---|---|
| Males:Cntl vs Females:Cntl | 1.14 | 0.15–2.14 | 0.0199 |

### RV/Tibial Length

ANOVA Summary:

| Source of Variation | p-value | Significant? |
|---|---|---|
| Interaction | ns | No |
| Sex | p<0.0001 | Yes |
| Diet_Ethanol | p=0.045 | Yes |

Tukey's Post-hoc Test:

| Comparison | Mean Difference | 95% CI of Difference | Adjusted P Value |
|---|---|---|---|
| Males:Cntl vs Females:Cntl | 0.39 | 0.14–0.64 | 0.0011 |
| Males:Cntl vs Females:EtOH | 0.49 | 0.27–0.71 | <0.0001 |
| Males:EtOH vs | 0.38 | 0.13–0.63 | 0.0014 |

| | | | |
|---|---|---|---|
| Females:Cntl | | | |
| Males:EtOH vs Females:EtOH | 0.48 | 0.26–0.70 | <0.0001 |

## Inflammatory Markers (qPCR)

### IL-6

ANOVA Summary:

| Source of Variation | p-value | Significant? |
|---|---|---|
| Interaction | p=0.027 | Yes |
| Sex | p<0.0001 | Yes |
| Diet_Ethanol | ns | No |

Tukey's Post-hoc Test:

| Comparison | Mean Difference | 95% CI of Difference | Adjusted P Value |
|---|---|---|---|
| Males:Cntl vs Males:EtOH | -1.74 | -3.48 to -0.01 | 0.0491 |

### IL-1β

ANOVA Summary:

| Source of Variation | p-value | Significant? |
|---|---|---|
| Interaction | p=0.003 | Yes |
| Sex | p<0.0001 | Yes |
| Diet_Ethanol | ns | No |

Tukey's Post-hoc Test:

| Comparison | Mean Difference | 95% CI of Difference | Adjusted P Value |
|---|---|---|---|
| Males:Cntl vs Males:EtOH | -2.35 | -3.91 to -0.80 | 0.0026 |
| Males:EtOH vs Females:Cntl | 2.33 | 0.77 to 3.88 | 0.0029 |
| Males:EtOH vs Females:EtOH | 2.29 | 0.73 to 3.84 | 0.0034 |

### IL-10

ANOVA Summary:

| Source of Variation | p-value | Significant? |
|---|---|---|
| Interaction | ns | No |
| Sex | ns | No |
| Diet_Ethanol | ns | No |

Tukey's Post-hoc Test:

| Comparison | Mean Difference | 95% CI of Difference | Adjusted P Value |
|---|---|---|---|

### Traf3ip2

ANOVA Summary:

| Source of Variation | p-value | Significant? |
|---|---|---|
| Interaction | ns | No |
| Sex | ns | No |
| Diet_Ethanol | ns | No |

Tukey's Post-hoc Test:

| Comparison | Mean Difference | 95% CI of Difference | Adjusted P Value |
|---|---|---|---|

## Fibrotic Markers

### Col1a1

ANOVA Summary:

| Source of Variation | p-value | Significant? |
|---|---|---|
| Interaction | ns | No |
| Sex | ns | No |
| Diet_Ethanol | ns | No |

Tukey's Post-hoc Test:

| Comparison | Mean Difference | 95% CI of Difference | Adjusted P Value |
|---|---|---|---|

### Col3a1

ANOVA Summary:

| Source of Variation | p-value | Significant? |
|---|---|---|
| Interaction | p<0.0001 | Yes |
| Sex | ns | No |
| Diet_Ethanol | ns | No |

Tukey's Post-hoc Test:

| Comparison | Mean Difference | 95% CI of Difference | Adjusted P Value |
|---|---|---|---|
| Males:Cntl vs Males:EtOH | 0.58 | 0.30–0.85 | <0.0001 |
| Males:EtOH vs Females:Cntl | -0.57 | -0.84 to -0.30 | 0.0001 |
| Males:EtOH vs Females:EtOH | -0.58 | -0.85 to -0.31 | <0.0001 |

### Col1a1/Col3a1

ANOVA Summary:

| Source of Variation | p-value | Significant? |
|---|---|---|
| Interaction | p=0.001 | Yes |
| Sex | ns | No |
| Diet_Ethanol | ns | No |

Tukey's Post-hoc Test:

| Comparison | Mean Difference | 95% CI of Difference | Adjusted P Value |
|---|---|---|---|
| Males:Cntl vs Males:EtOH | -1.88 | -2.95 to -0.81 | 0.0007 |
| Males:EtOH vs Females:Cntl | 1.89 | 0.82–2.96 | 0.0006 |
| Males:EtOH vs Females:EtOH | 1.89 | 0.82–2.96 | 0.0006 |

### Larp6

ANOVA Summary:

| Source of Variation | p-value | Significant? |
|---|---|---|
| Interaction | p=0.033 | Yes |
| Sex | ns | No |
| Diet_Ethanol | ns | No |

Tukey's Post-hoc Test:

| Comparison | Mean Difference | 95% CI of Difference | Adjusted P Value |
|---|---|---|---|
| Males:Cntl vs Males:EtOH | -1.81 | -3.50 to -0.12 | 0.0334 |
| Males:EtOH vs Females:Cntl | 1.80 | 0.21–3.40 | 0.0241 |
| Males:EtOH vs Females:EtOH | 1.81 | 0.22–3.41 | 0.0234 |

## Mitochondrial Function

### ROS Levels

ANOVA Summary:

| Source of Variation | p-value | Significant? |
|---|---|---|
| Interaction | p=0.364 | No |
| Sex | p=0.037 | Yes |
| Diet_Ethanol | p=0.687 | No |

Tukey's Post-hoc Test:

| Comparison | Mean Difference | 95% CI of Difference | Adjusted P Value |
|---|---|---|---|
| Males:Cntl vs Males:EtOH | -0.037 | -0.152 to 0.077 | 0.8001 |

### ATP Levels

ANOVA Summary:

| Source of Variation | p-value | Significant? |
|---|---|---|
| Interaction | p=0.309 | No |
| Sex | p=0.176 | No |
| Diet_Ethanol | p=0.237 | No |

Tukey's Post-hoc Test:

| Comparison | Mean Difference | 95% CI of Difference | Adjusted P Value |
|---|---|---|---|

## Mitochondrial Function – Respiration States

### State 2

ANOVA Summary:

| Source of Variation | p-value | Significant? |
|---|---|---|
| Interaction | p=0.021 | Yes |
| Sex | p=0.004 | Yes |
| Diet_Ethanol | ns | No |

Tukey's Post-hoc Test:

| Comparison | Mean Difference | 95% CI of Difference | Adjusted P Value |
|---|---|---|---|
| Males:Cntl vs Females:EtOH | 23.16 | 3.33 to 43.00 | 0.0187 |

### State 3

ANOVA Summary:

| Source of Variation | p-value | Significant? |
|---|---|---|
| Interaction | p=0.014 | Yes |
| Sex | p<0.0001 | Yes |
| Diet_Ethanol | ns | No |

Tukey's Post-hoc Test:

| Comparison | Mean Difference | 95% CI of Difference | Adjusted P Value |
|---|---|---|---|
| Males:Cntl vs Females:Cntl | 71.70 | 23.69 to 119.7 | 0.0025 |

### State 3u

ANOVA Summary:

| Source of Variation | p-value | Significant? |
|---|---|---|
| Interaction | p=0.028 | Yes |
| Sex | p=0.001 | Yes |
| Diet_Ethanol | ns | No |

Tukey's Post-hoc Test:

| Comparison | Mean Difference | 95% CI of Difference | Adjusted P Value |
|---|---|---|---|
| Males:Cntl vs Females:EtOH | 67.46 | 7.22 to 127.7 | 0.0249 |
| Males:EtOH vs Females:Cntl | 103.0 | 42.04 to 163.9 | 0.0007 |
| Males:EtOH vs Females:EtOH | 121.7 | 54.96 to 188.4 | 0.0003 |

### State 4o

ANOVA Summary:

| Source of Variation | p-value | Significant? |
|---|---|---|
| Interaction | ns | No |
| Sex | ns | No |
| Diet_Ethanol | ns | No |

Tukey's Post-hoc Test:

| Comparison | Mean Difference | 95% CI of Difference | Adjusted P Value |
|---|---|---|---|

### State 4u

ANOVA Summary:

| Source of Variation | p-value | Significant? |
|---|---|---|
| Interaction | ns | No |
| Sex | ns | No |
| Diet_Ethanol | ns | No |

Tukey's Post-hoc Test:

| Comparison | Mean Difference | 95% CI of Difference | Adjusted P Value |
|---|---|---|---|
