# Supplementary material for: Female Cardioprotection in a Mouse Model of Alcohol-Associated Cardiomyopathy
Source: Cells. 2025 Oct 27;14(21):1682. doi: 10.3390/cells14211682 (PMC12607819; doi:10.3390/cells14211682)

Supplementary Information

for

Female Cardioprotection in a Mouse Model of Alcohol-Associated Cardiomyopathy

Joshua M. Edavettal, Meagan Donovan, Nicholas R. Harris, Xavier R. Chapa-Dubocq , Keishla M. Rodríguez-Graciani , Janos Paloczi , Liz Simon , Bysani Chandrasekar and Jason D. Gardner

Supplementary Figure S1.

Full-length blots corresponding to Figure 6A & 6C (Collagen I and alpha-Smooth Muscle Actin (a-SMA). Total protein stain are shown for each blot. After total protein staining, the blots were cut to allow for separate western blot of Collagen I and a-SMA. Collagen I has a much higher molecular weight (> 75 kDa) than a-SMA (~42 kDa). Regions of interest for quantification are shown on the right side of the blot images. C = Control; E = Ethanol.

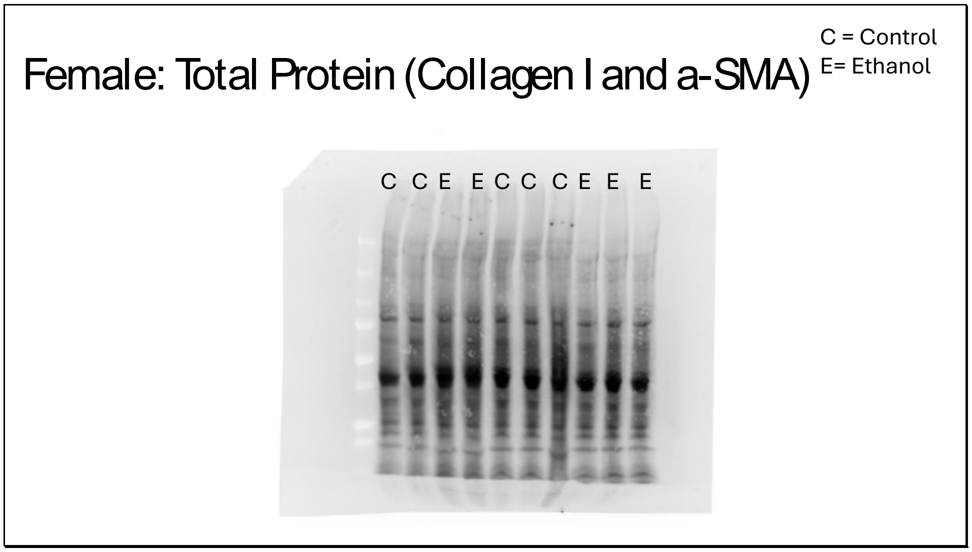

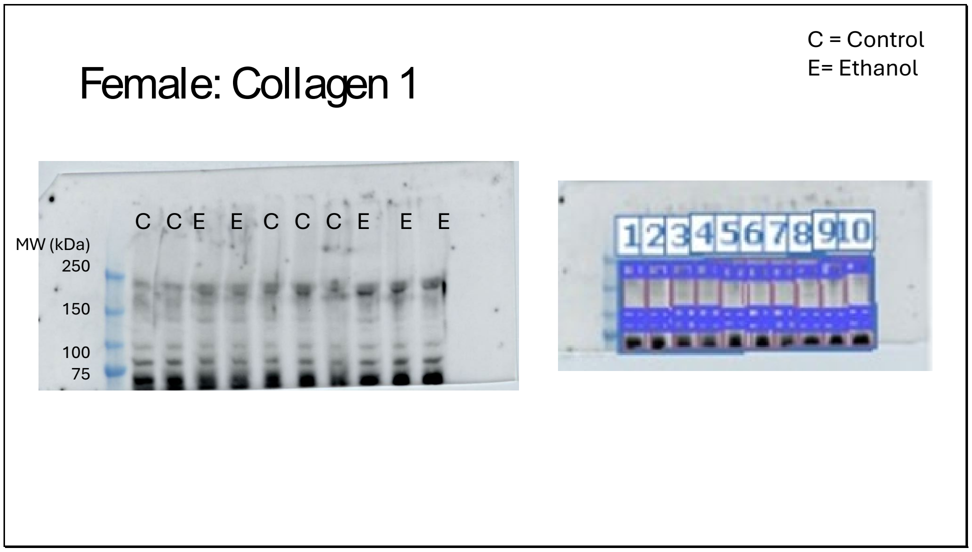

C = Control
E= Ethanol

Female: alpha Smooth Muscle Actin

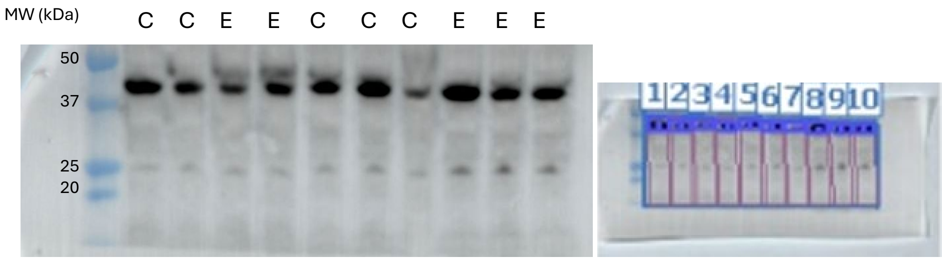

C = Control
E= Ethanol

Male: Total Protein (Collagen I and a-SMA)

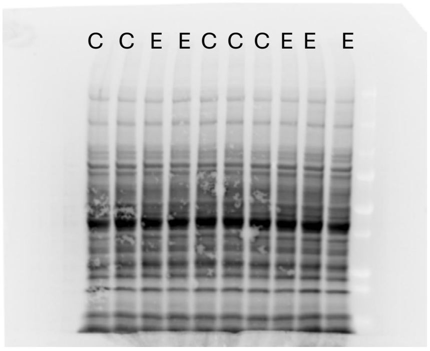

C = Control
E= Ethanol

Male: Collagen 1

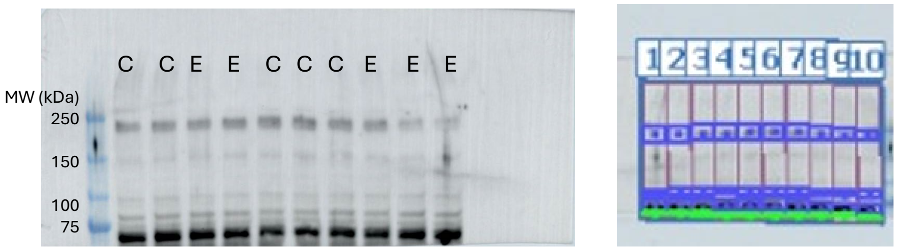

Male: alpha Smooth Muscle Actin

C = Control
E= Ethanol

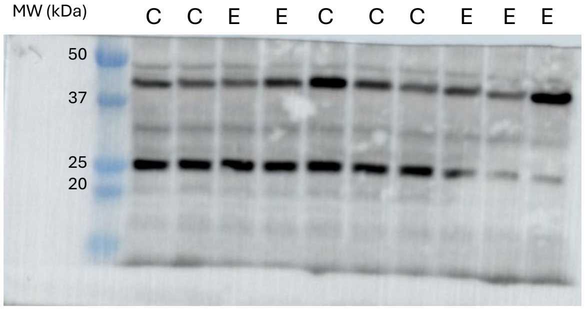

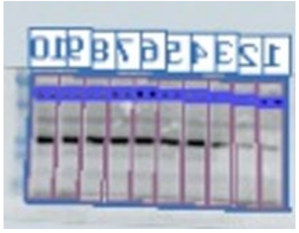

Supplementary Figure S2.

Full-length blots corresponding to Figure 6B (Collagen III). Total protein stain are shown for each blot. Regions of interest for quantification are shown on the right side of the blot images. C = Control; E = Ethanol.

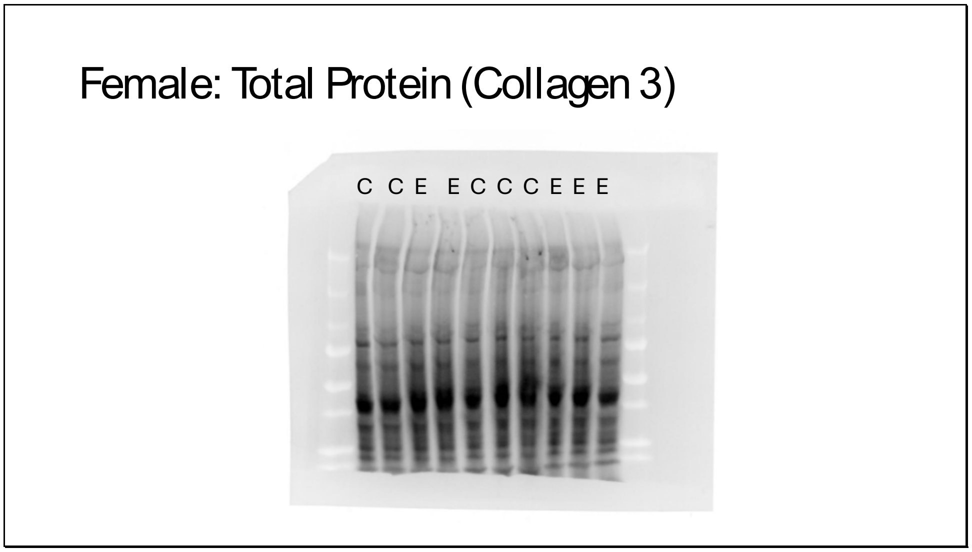

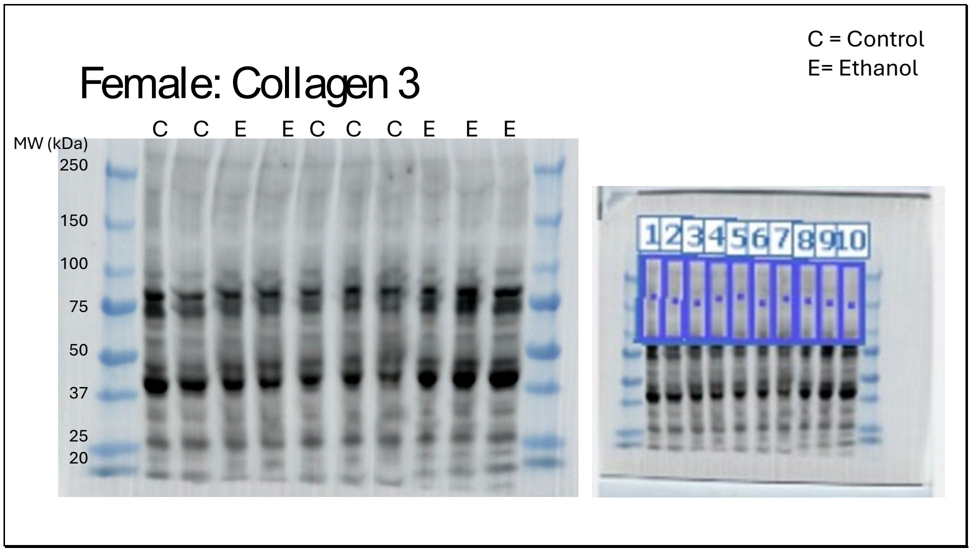

Male: Total Protein (Collagen 3)

C = Control
E= Ethanol

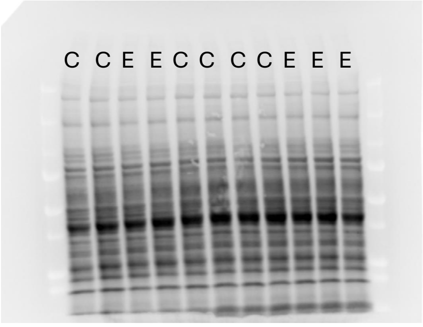

Male: Collagen 3

C = Control
E= Ethanol

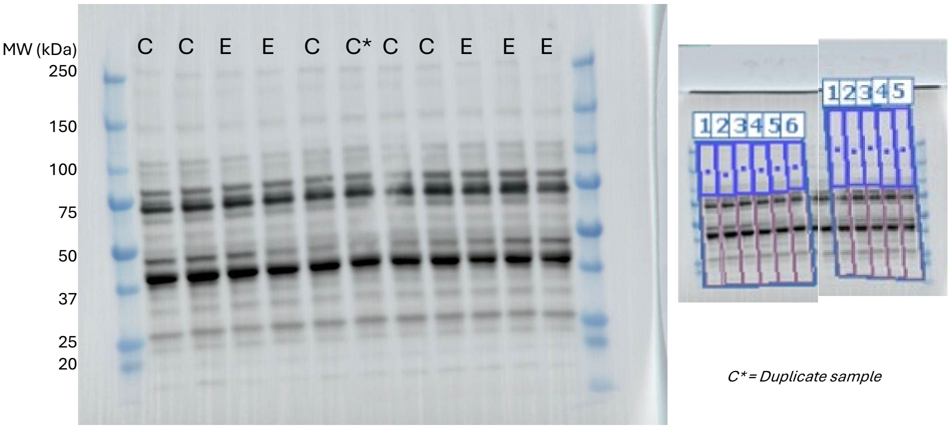

C = Duplicate sample*

Note: All blots are shown as unedited images corresponding to the quantifications presented in the main figures. Quantified areas and total protein stains are marked for transparency and reproducibility.

Supplementary Figure S3.

Alternative quantification of Collagen I and III (Figure 6A & 6B) for individual bands ranging from ~80-230 kDa. Data are analyzed by 2-way ANOVA with Tukey's post hoc (ns = P > 0.05, *P < 0.05; C/Cntl = Control; E/EtOH = Ethanol).

COLLAGEN I:

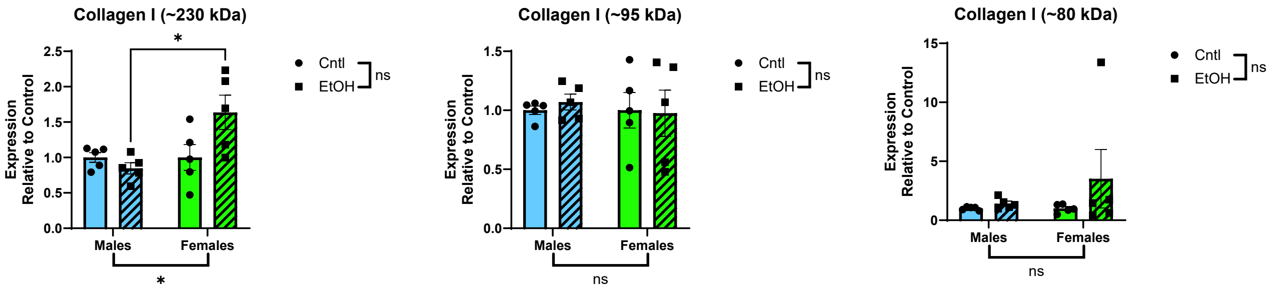

Collagen I (Males)

Molecular Weight (kDa)	Lane (Group)	Band	Volume	Normalized to Total Protein	Relative to Control
~230	1 (E)	1	34818	0.020445769	0.781700245
	2 (E)	1	28492	0.015577792	0.595583558
	3 (E)	1	54736	0.028207614	1.078457765
	4 (C)	1	59564	0.028143566	1.076009038
	5 (C)	1	66936	0.029186857	1.115897044
	6 (C)	1	63862	0.029483882	1.127253152
	7 (E)	1	57896	0.024160561	0.923727351
	8 (E)	1	53249	0.022338857	0.854078416
	9 (C)	1	50775	0.020750101	0.793335733
	10 (C)	1	49383	0.023213147	0.887505033
~95	1 (E)	2	26375	0.015487885	1.24691672
	2 (E)	2	24266	0.013267258	1.068135896
	3 (E)	2	28644	0.01476138	1.188426433
	4 (C)	2	26139	0.012350491	0.994327809
	5 (C)	2	29732	0.012964378	1.043751345
	6 (C)	2	27975	0.01291553	1.039818623
	7 (E)	2	27664	0.011544455	0.929434465
	8 (E)	2	27140	0.01138569	0.916652415
	9 (C)	2	26226	0.010717719	0.862874618
	10 (C)	2	27989	0.013156608	1.059227604
~80	1 (E)	3	254823	0.149636747	2.126888473
	2 (E)	3	198373	0.108458984	1.541601029
	3 (E)	3	176998	0.091214031	1.296486832
	4 (C)	3	160249	0.075716512	1.076210087
	5 (C)	3	128258	0.055925779	0.794910989
	6 (C)	3	137690	0.063568878	0.903547543

	7 (E)	3	165400	0.06902302	0.981070947
	8 (E)	3	186305	0.078158103	1.11091407
	9 (C)	3	197899	0.080874925	1.149530106
	10 (C)	3	161016	0.07568775	1.075801275

Collagen I (Females)

Molecular Weight (kDa)	Lane (Group)	Band	Volume	Normalized to Total Protein	Relative to Control
~230	1 (C)	1	11282	0.006096302	0.472187452
	2 (C)	1	20148	0.010301493	0.797899361
	3 (E)	1	33777	0.015224171	1.17918404
	4 (E)	1	28325	0.012884828	0.997990844
	5 (C)	1	27158	0.012606936	0.976466817
	6 (C)	1	42723	0.019895463	1.540997716
	7 (C)	1	35925	0.015653643	1.212448655
	8 (E)	1	52973	0.028827949	2.232861028
	9 (E)	1	48983	0.026870375	2.081237607
	10 (E)	1	38070	0.021894309	1.695817808
~95	1 (C)	2	5977	0.003229711	1.428060237
	2 (C)	2	5159	0.002637751	1.166317058
	3 (E)	2	2811	0.001266991	0.560217007
	4 (E)	2	2375	0.001080369	0.477699929
	5 (C)	2	2507	0.001163767	0.514575333
	6 (C)	2	4833	0.002250656	0.995157851
	7 (C)	2	4650	0.00202615	0.895889521
	8 (E)	2	5675	0.00308834	1.365550971
	9 (E)	2	4403	0.002415333	1.067972069
	10 (E)	2	5526	0.003178039	1.405212874
~80	1 (C)	3	13832	0.007474211	1.321291011
	2 (C)	3	10625	0.005432468	0.960351607
	3 (E)	3	7771	0.003502591	0.619188071
	4 (E)	3	4993	0.002271278	0.401516464
	5 (C)	3	6042	0.002804739	0.495821757
	6 (C)	3	10334	0.004812389	0.850734147
	7 (C)	3	17809	0.007759937	1.371801479
	8 (E)	3	15177	0.008259336	1.460085247
	9 (E)	3	18023	0.009886793	1.747787065
	10 (E)	3	19641	0.07568775	13.38007947

COLLAGEN III:

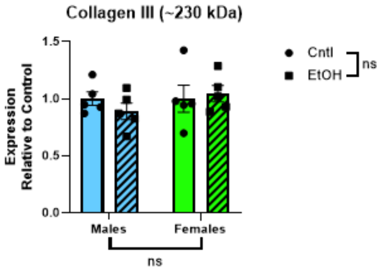

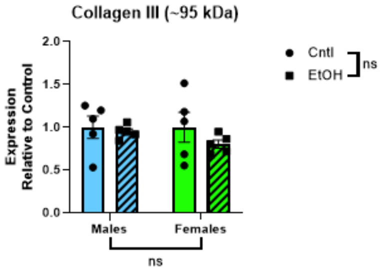

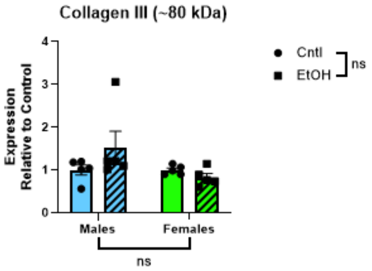

Collagen III (Males)

Molecular Weight (kDa)	Lane (Group)	Band	Volume	Normalized to Total Protein	Relative to Control
~230	1 (C)	1	4754	0.003009082	0.92742411
	2 (C)	1	5285	0.002828961	0.871909433
	3 (E)	1	5671	0.00321657	0.991373836
	4 (E)	1	4925	0.002676502	0.824920407
	5 (C)	1	7254	0.003929797	1.211196181
	7 (C)	1	7956	0.003377511	1.040977008
	8 (C)	1	7740	0.003077442	0.948493269
	9 (E)	1	5373	0.002171786	0.669362496
	10 (E)	1	8881	0.003546072	1.092929132
	11 (E)	1	6392	0.002822322	0.869863147
~95	1 (C)	2	20218	0.012797142	1.197812537
	2 (C)	2	24995	0.013379353	1.252307505
	3 (E)	2	17864	0.010132395	0.9483922
	4 (E)	2	18046	0.00980714	0.917948302
	5 (C)	2	21641	0.011723839	1.097351405
	7 (C)	2	13353	0.005668665	0.530587079
	8 (C)	2	24773	0.009849802	0.921941475
	9 (E)	2	22271	0.009002017	0.842588854
	10 (E)	2	25897	0.010340349	0.967856668
	11 (E)	2	25540	0.011276924	1.05552012
~80	1 (C)	3	46935	0.029707877	1.200000346
	2 (C)	3	54579	0.029215112	1.180095905
	3 (E)	3	52665	0.029871394	1.206605328
	4 (E)	3	55077	0.029931721	1.209042127
	5 (C)	3	46458	0.025168252	1.016629706
	7 (C)	3	32658	0.013864096	0.560017105
	8 (C)	3	64958	0.02582745	1.043256939
	9 (E)	3	62120	0.025109125	1.014241382

	10 (E)	3	68110	0.027195472	1.098515925
	11 (E)	3	56982	0.07568775	3.057280911

Collagen III (Females)

Molecular Weight (kDa)	Lane	Band	Volume	Normalized to Total Protein	Relative to Control
~230	1 (C)	1	44595	0.052087348	0.698260039
	2 (C)	1	96204	0.106219665	1.423934016
	3 (E)	1	87379	0.06933328	0.929451404
	4 (E)	1	100508	0.076009293	1.018947084
	5 (C)	1	73201	0.070437253	0.944250781
	6 (C)	1	75365	0.072971816	0.978228013
	7 (C)	1	75429	0.071263506	0.955327152
	8 (E)	1	101035	0.096011122	1.287082793
	9 (E)	1	91446	0.082222376	1.102236941
	10 (E)	1	68739	0.066098624	0.88608903
~95	1 (C)	2	22333	0.026085138	0.68440394
	2 (C)	2	19058	0.021042102	0.55208821
	3 (E)	2	41469	0.032904723	0.863331517
	4 (E)	2	47760	0.036118556	0.947653853
	5 (C)	2	42328	0.040729882	1.068642647
	6 (C)	2	46489	0.045012761	1.181013906
	7 (C)	2	61071	0.057698412	1.513851298
	8 (E)	2	28782	0.02735084	0.717612545
	9 (E)	2	28865	0.025953556	0.68095157
	10 (E)	2	31799	0.030577549	0.802272725
~80	1 (C)	3	124032	0.144870456	1.146407384
	2 (C)	3	112885	0.1246373	0.986295793
	3 (E)	3	116837	0.092707544	0.73362517
	4 (E)	3	127586	0.096487062	0.763533735
	5 (C)	3	118278	0.113812345	0.900634375
	6 (C)	3	117874	0.114130961	0.903155688
	7 (C)	3	142250	0.13439438	1.063506759
	8 (E)	3	118141	0.112266541	0.888401921
	9 (E)	3	161105	0.14485528	1.146287287
	10 (E)	3	157628	0.07568775	0.598941963

Supplementary Figure S4.

Fractional shortening (FS) and ejection fraction (EF) assessed by ultrasound echocardiography indicated no interaction and no main effects of sex or ethanol after 30 d of chronic+binge ethanol feeding. Data analyzed by 2-way ANOVA. Cntl = control; EtOH = ethanol-fed.

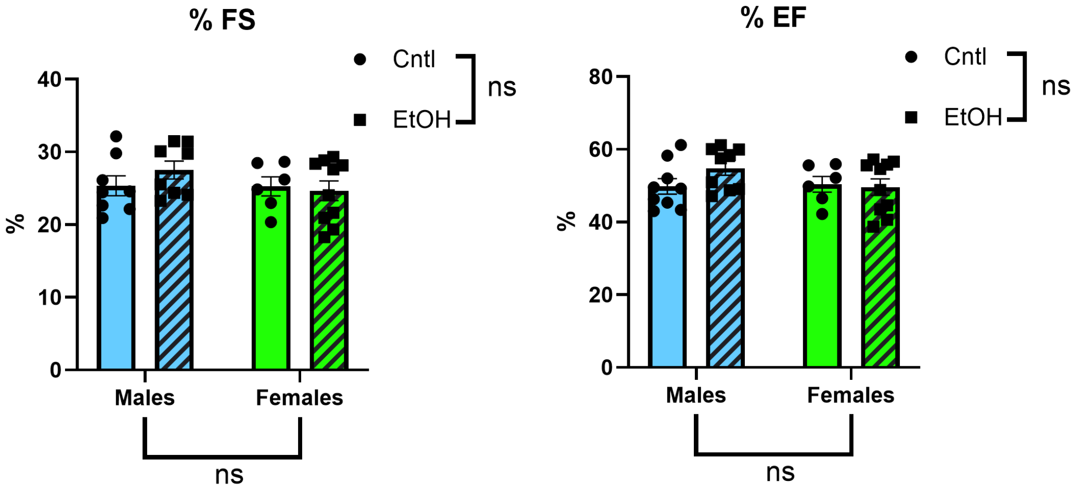

Supplement: Supplementary file 1 [file cells-14-01682-s001.zip › Supplemental_FigureS1-4_WBandEcho.pdf]
